# Flower-like SnO_2_ Nanoparticle Biofabrication Using *Pometia pinnata* Leaf Extract and Study on Its Photocatalytic and Antibacterial Activities

**DOI:** 10.3390/nano11113012

**Published:** 2021-11-10

**Authors:** Is Fatimah, Gani Purwiandono, Habibi Hidayat, Suresh Sagadevan, Sheikh Ahmad Izaddin Sheikh Mohd Ghazali, Won-Chun Oh, Ruey-An Doong

**Affiliations:** 1 Department of Chemistry, Faculty of Mathematics and Natural Sciences, Universitas Islam Indonesia, Kampus Terpadu UII, Jl. Kaliurang Km 14, Sleman, Yogyakarta 55584, Indonesia; gani_purwiandono@uii.ac.id (G.P.); habibihidayat13@uii.ac.id (H.H.); 2 Nanotechnology& Catalysis Research Centre, University of Malaya, Kuala Lumpur 50603, Malaysia; drsureshnano@gmail.com; 3 Faculty of Applied Sciences, Universiti Teknologi MARA Cawangan Negeri Sembilan, Kampus Kuala Pilah, Kuala Pilah 72000, Malaysia; sheikhahmadizaddin@uitm.edu.my; 4 Department of Advanced Materials Science and Engineering, Hanseo University, Seosan-si 356-706, Chungnam, Korea; 5 Institute of Analytical and Environmental Sciences, National Tsing Hua University, 101, Sec 2, Kuang Fu Road, Hsinchu 30013, Taiwan; radoong@mx.nthu.edu.tw

**Keywords:** antibacterial, green synthesis, nanoparticles, photocatalyst, tin oxide

## Abstract

The present study reported biofabrication of flower-like SnO_2_ nanoparticles using *Pometia pinnata* leaf extract. The study focused on the physicochemical characteristics of the prepared SnO_2_ nanoparticles and its activity as photocatalyst and antibacterial agent. The characterization was performed by XRD, SEM, TEM, UV-DRS and XPS analyses. Photocatalytic activity of the nanoparticles was examined on bromophenol blue photooxidation; meanwhile, the antibacterial activity was evaluated against *Klebsiella pneumoniae*, *Escherichia coli Staphylococcus aureus* and *Streptococcus pyogenes*. XRD and XPS analyses confirmed the single tetragonal SnO_2_ phase. The result from SEM analysis indicates the flower like morphology of SnO_2_ nanoparticles, and by TEM analysis, the nanoparticles were seen to be in uniform spherical shapes with a diameter ranging from 8 to 20 nm. SnO_2_ nanoparticles showed significant photocatalytic activity in photooxidation of bromophenol blue as the degradation efficiency reached 99.93%, and the photocatalyst exhibited the reusability as the degradation efficiency values were insignificantly changed until the fifth cycle. Antibacterial assay indicated that the synthesized SnO_2_ nanoparticles exhibit an inhibition of tested bacteria and showed a potential to be applied for further environmental and medical applications.

## 1. Introduction

Recently, green processing in chemical reactions and environmental applications have increasingly attracted attention. Within these schemes, studies on nanotechnology, including the exploration of synthesis, characterization, modification and application, were developed [1,2]. The enhanced physicochemical properties with more intense activities for efficiencies were achieved by nanomaterials in many areas of applications such as in wastewater treatment, biomedical, optics, sensor, antibacterial and electrochemical applications [3,4,5]. For example, metal oxide nanoparticles have been widely used as more effective photocatalysts and antibacterial agents compared to the bulk form. The size and morphology of the low dimensional metal oxide nanostructures govern the unique chemical and optical properties, and are intensively studied and give opportunity for better effectiveness. Metal oxide nanoparticles such as TiO_2_, ZnO, SnO_2_ and ZrO_2_ and their combination with other metal/metal oxides were demonstrated to be effective photocatalysts in water treatment processes, including for pharmaceutics and dye-containing wastewaters [6,7,8,9]. The complete removal of organic dyes under mild conditions has been demonstrated with these nanoparticle photocatalysts. The more effective interaction between light as a photon source and the nanoparticle surface facilitated the faster reduction–oxidation reactions which take place at the particle’s interfaces [10,11,12,13,14]. By this mechanism, oxidation can also occur concerning bacteria or viruses, leading to potential application of semiconductor metal oxide nanoparticles as antibacterial, antiviral or antifungal agents for medical and sanitation technologies. 

From the perspective of green synthesis, the utilization of plant extracts along with the intensified process was also considered a more reliable, low-cost and eco-friendly method. Amongst the different biomaterials, plant extracts consisting of leaves, bulbs, petals or fruits have been employed for preparing metal oxide nanoparticles [15,16]. The presence of secondary metabolites such as flavonoids, polyphenols, vitamins and proteins act as bioreductors and capping agents, which contribute to stabilizing the nanoparticles and give benefits for medical applications. Among many photoactive metal oxide nanoparticles, SnO_2_ nanoparticles (SnO_2_ NPs) have been widely studied for such purposes, and have exhibited highly photocatalytic activity with a band gap energy of about 3.2–3.6 eV. SnO_2_ also offers and gives higher electron mobility (100–200 cm^2^V^−1^s^−1^) compared to other semiconductors such as TiO_2_, leading to faster photo generated electron transport during the photocatalysis mechanism [17]. From several studies on the use of plant extracts in the SnO_2_ NP synthesis, it can be concluded that different plant species gave the specific character and morphology of the nanoparticles, and influence the physicochemical character and activity [18]. Matoa or *Pometia pinnata* is an endemic plant in Southeast Asia, including Indonesia, especially in Papua Island. The leaf of *Pometia pinnata* has been utilized in traditional medical applications, and further analysis has revealed that the activities in medical applications are in correlation with the high content of flavonoids, tannins, triterpenoids, glycosides and saponins [17,19,20]. These compounds have been proven to be effective as bioreductors for the plant extract-mediated synthesis of silver NPs and in the synthesis of reduced graphene oxide [21,22], but, to our knowledge, utilization for SnO_2_ NP synthesis has not been reported yet. As exploration for the synthesis, characterization and application of SnO_2_ NPs, this research aimed to study the physicochemical character of the SnO_2_ NPs and their activity as photocatalyst and antibacterial agent. Bromophenol blue (BPB) was chosen as the model of dye compounds, since it is highly consumed in many industries. In addition, the persistence and toxic character of BPB requires attention concerning being treated; it will be particularly representative for photocatalytic degradation. For antibacterial activity assay, the inhibitory activity of SnO_2_ NPs was examined against *Klebsiella pneumoniae* and *Escherichia coli*, which were representative of gram-negative bacteria, and *Staphylococcus aureus* and *Streptococcus pyogenes* as gram-positive bacteria. 

## 2. Materials and Methods

### 2.1. Materials

The SnCl_2_·2H_2_O, BPB and ethanol with 99% purity were purchased from Sigma-Aldrich Chemical Company (Darmstadt, Germany). Double-distilled water was used in all experiments.

### 2.2. Extraction of Pometia pinnata Leaves

Fresh *Pometia pinnata* leaves were harvested from rural farms in Sleman, Yogyakarta Province, Indonesia, and then washed several times with distilled water to remove dirt. Afterwards, the leaves were air dried for a week at ambient temperature to reach the appropriate moisture. The dried leaves were ground into fine powders using a mortar and pestle; furthermore, 20 g of the dried leaves were refluxed with 100 mL of distilled water for 4 h. The *Pometia pinnata* leaf extract (PPE) was obtained by filtering the result using Whatman Grade 1 filter paper. To maintain the condition of the PPE, the PPE was kept at a temperature of 5 °C in a refrigerator.

### 2.3. Synthesis of SnO_2_ NPs 

**Figure 1:**
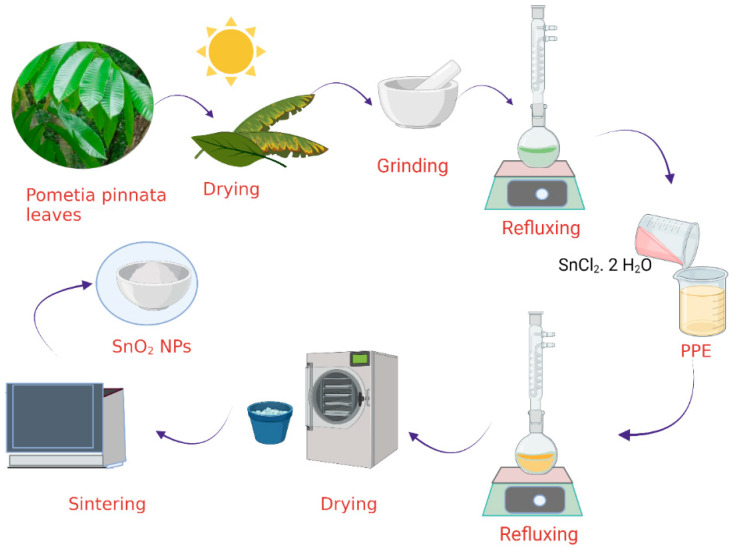
Schematic representation of SnO_2_ NP synthesis.

The synthesis of SnO_2_ NPs was performed by mixing 2 g of tin chloride (SnCl_2_·2H_2_O) with 50 mL of PPE, followed by the addition of 50 mL of distilled water. The mixture was then refluxed for an hour, and the monitoring of nanoparticle formation was performed by UV-visible spectrophotometry. As the formation of the nanoparticles was confirmed by UV visible spectroscopy, the precipitate was obtained by evaporating water in an oven at a constant temperature of 60 °C. The powder obtained from these steps was then sintered at 500 °C for 2 h to get SnO_2_ NPs. Figure 1 displays the schematic representation of SnO_2_ NP synthesis. 

### 2.4. Characterization of SnO_2_ NPs 

X-ray diffraction (XRD) measurement was carried out using a Shimadzu X6000 diffractometer with Ni-filtered Cu-Kα radiation (*λ* =1.5406 Å) operated at a voltage of 40 kV and current of 30 mA. The scanning was taken from 10 to 70°. The crystalline size of the NPs was measured based on the Scherer equation:

*d* = *kλ*/*B*cos*θ* (1)

where *d* is the mean crystalline size of the NPs, *λ* is the wavelength of radiation (1.5406 Å), *θ* is the selected angle and *B* is the full width at half maximum (FWHM) intensity of the selected reflection. 

The morphology of prepared nanoparticles was investigated on a Phenom-X field-emission scanning electron microscope (FE-SEM); meanwhile, the elemental composition of the SnO_2_ NPs was determined by X-ray Fluorescence spectroscopy (XRF) using the Shimadzu EDX-7000 instrument. Transmission electron microscopy (TEM) images and high-resolution TEM (HR-TEM) images were obtained with a JEOL TEM 2010 transition electron microscope operated at 200 kV. The sample was collected on a holey carbon grid. XPS analysis was performed on a V.G. Scientific ESKALAB MKII instrument with a monochromatic Al *K*_α_ radiation with photon energy of 1486.6 ± 0.2 eV. Prior to being analyzed, the sample was slightly pressed into a small size and then degassed to achieve a dynamic vacuum below 10^−8^ Pa.

### 2.5. Photocatalytic Activity of SnO_2_ NPs 

To verify the photocatalytic performances of SnO_2_ NPs, experiments on photocatalytic oxidation (herein called photooxidation) of BPB were conducted under UV and visible light illumination. A 20 watt Philips UV light lamp was employed as the UV light source, and a 20 watt Philip Xenon lamp was utilized as the visible light source. The light intensities of UV light and Xenon lamps were 39.99 MW/Cm^2^ and 31.22 MW/Cm^2^, respectively; the radiometer was VLX-3W. The experiments were executed in a batch photocatalytic reactor equipped with a water-jacketed chamber to stabilize the temperature. In particular, 0.25 g of SnO_2_ NPs was dispersed in 100 mL of 20 ppm BPB solution, and about 1 mL of 5 × 10^−3^ M of H_2_O_2_ was then added. The suspension was stirred in the dark for 15 min before being exposed to light, and the samples of supernatant were then collected sequentially for colorimetric analysis using UV-visible spectrophotometry. 

The degradation efficiency of the photocatalysis was calculated based on the initial concentration and the concentration at the time of sampling with reference to the following equation (Equation (2)):

(2) $$\mathrm{Degradation}\;\mathrm{efficiency}\;\left(\mathrm{DE}\right)\left(\%\right)=100\times \left(\frac{C_{0}-C_{t}}{C_{0}}\right)$$

where *C*_0_ and *C_t_* are initial concentration and concentration of BPB at time *t*. 

### 2.6. Antibacterial Assay of SnO_2_ NPs 

Antibacterial activity of the SnO_2_ NPs was evaluated by disc diffusion method. The tested bacterial strains were *S. aureus* (ATCC 25923), *S. pyogenes* (ATCC 19615), *K. pneumoniae* (ATCC 13883) and *E. coli* (ATCC 11303). The medium for bacterial growth was prepared by suspending nutrient agar in distilled water and was autoclaved before use. Bacterial culture was evenly spread throughout the petri plate and a 6 mm sterile filter disc loaded with 100 μg/100 mL SnO_2_ NPs solution. 

## 3. Results

### 3.1. SnO_2_ NP Synthesis and Characterization

The formation of SnO_2_ NPs was initially monitored by the identification of surface plasmon resonance (SPR) using UV-visible spectrophotometry analysis on a HITACHI U-2010 instrument. 

**Figure 2:**
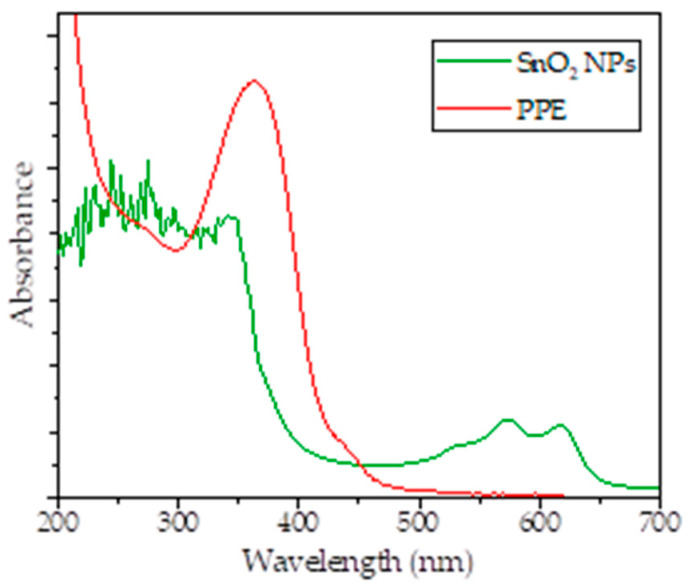
UV-visible spectra of produced SnO_2_ NPs in comparison with PPE.

**Figure 3:**
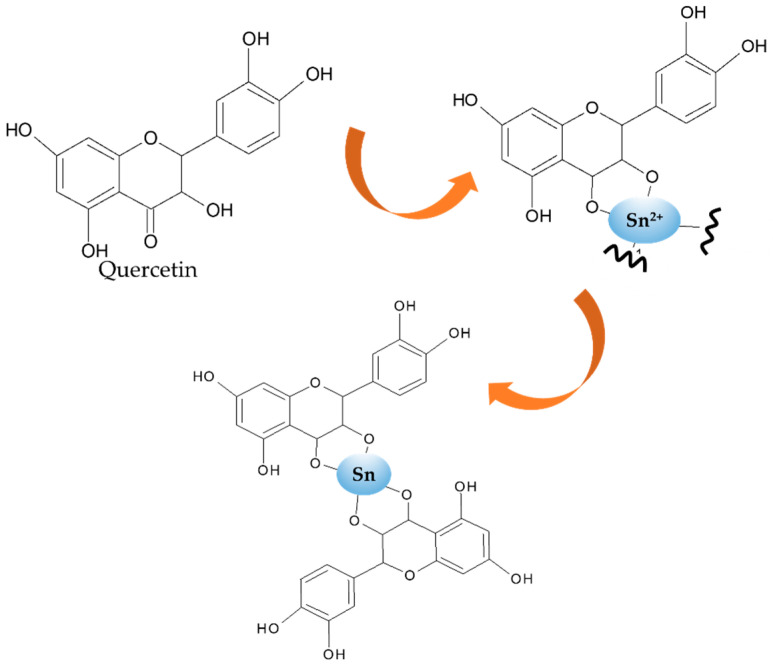
Mechanism of Sn^2+^ bioreduction by quercetin in PPE.

Figure 2 shows that the bioreduction occurred as the change of peaks recorded values of from 200 to 800 nm. The PPE showed peaks at the range of 200–300 nm and 500–600 nm, indicating the presence of secondary metabolites which contain aromatic structures. The spectrum of SnO_2_ NPs shows the characteristic surface plasmon resonance (SPR) absorption band at 396 nm. This wavelength is consistent with the SPR absorption band of Sn NPs in previous literature mentioning the range of 295–400 nm. The disappearing peaks at the range of 500–600 nm is an indication of the reduction of some secondary metabolite. A previous study identified some secondary metabolites such as kaempferol-3-O-rhamnoside, glycolipid, epicatechin, quercetin-3-O-rhamnoside, steroid glycosides, and other saponin compounds as the components of PPE [23,24]; the reduction of Sn^2+^ possibly involves the functional groups of those compounds. Taking quercetin as one of the bioreductors, the reduction mechanism is as presented in Figure 3. 

Moreover, other phenolic compounds work as singlet oxygen quenchers, hydrogen donators, metal chelating and reducing agents which furthermore act as capping agents for the nanoparticles [25].

**Figure 4:**
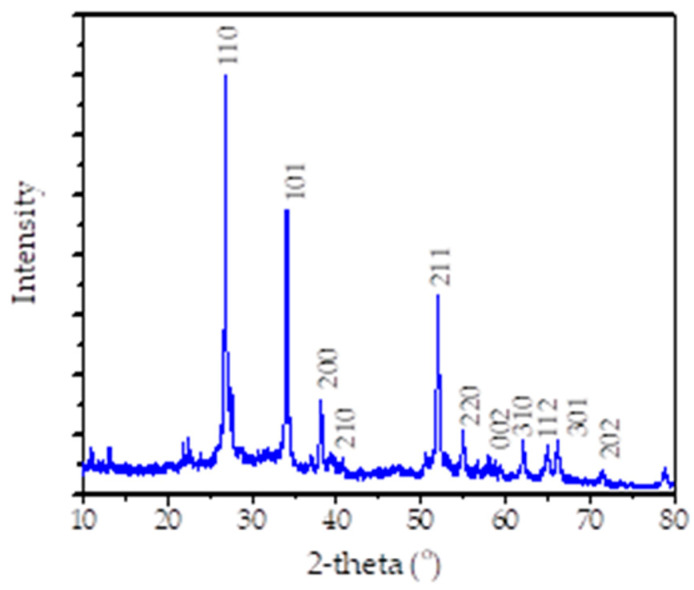
XRD pattern of SnO_2_ NPs.

**Figure 5:**
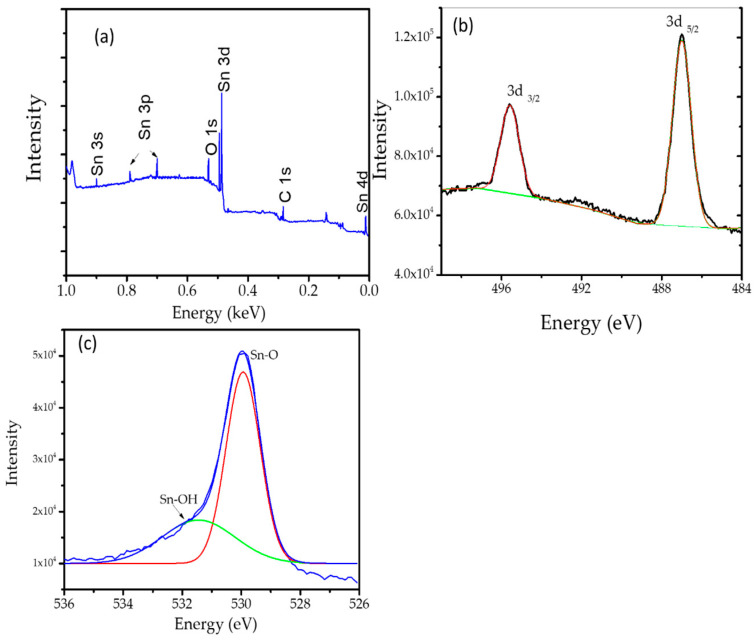
(**a**) Survey scan spectrum of SnO_2_ NPs. (**b**) Spectrum of 3d. (**c**) Deconvoluted spectrum of O1s.

Results from the XRD analysis presented in Figure 4 exhibit the peaks which are associated with the (110), (101), (200), (210), (211), (220), (002), (310), (112), (301) and (202) planes, respectively. All diffraction peaks coincide strongly with the planes of standard tetragonal rutile SnO_2_, which refers to JCPDS 41-1445, and there is no other peak detected, indicating the single phase of the nanoparticles [26,27]. Based on the Scherer equation, the average crystallite size of SnO_2_ NPs calculated from (110), (101) and (211) is about 18.2 nm. Complete oxide formation was observed by XPS analysis, and the spectra are presented in Figure 5. 

The survey scan spectrum (Figure 5a) shows the major peaks of C, O and Sn, clearly indicating the successful fabrication of SnO_2_ by using PPE as the reducing agents. The deconvoluted Sn 3d spectrum (Figure 5b) shows two peaks at 487.1 and 495.9 eV, which are assigned as the Sn 3d_5/2_ and Sn 3d_3/2_ spin-orbit peaks, indicating the chemical state of the SnO_2_ NPs as Sn^4+^. In addition, the O1s spectrum can be deconvoluted into two peaks centered at 530.6 and 531.8 eV, associated with the Sn-O and Sn-OH, respectively (Figure 5c). These peaks are ascribed as the oxygen-containing functional groups bound to the SnO_2_ NPs as the caping agent [18,28,29]. 

**Figure 6:**
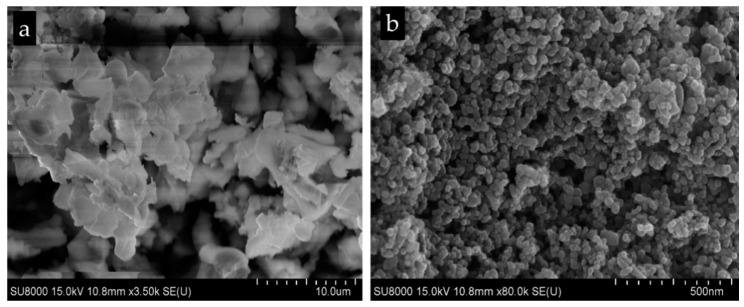
(**a**). Flower-like form of FE-SEM images of the SnO_2_ NPs. (**b**) FE-SEM images of the SnO_2_ NPs with higher magnification.

Figure 6a shows the flower-like FE-SEM image of the SnO_2_ NPs, while Figure 6b represents the higher magnification of the image. By the higher magnification, the nanoparticle facets of the of the structures are smooth and discriminable in uniform spherical shapes. 

**Figure 7:**
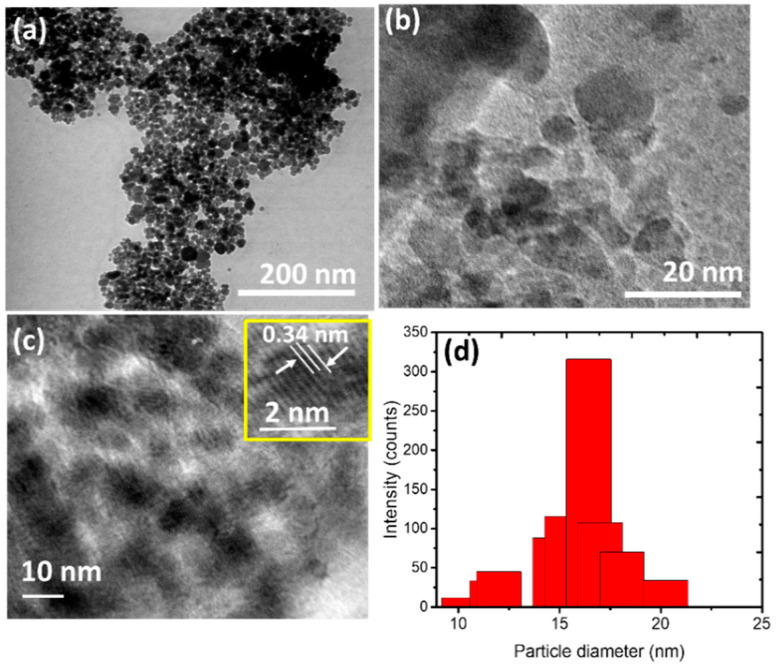
(**a**,**b**) TEM images of the SnO_2_ NPs at different magnifications. (**c**) HR-TEM image of the particles (**d**). Particle size distribution of nanoparticles.

Moreover, the TEM and HR-TEM investigations represent further insight into the morphologies and the structural features of SnO_2_ NPs. It can be seen from the TEM profile (Figure 7a–c) that the irregular spherical forms have diameters ranging from 8 to 18 nm. In addition, HR-TEM images (Figure 7c) represent the clear lattice fringes with a width of 0.34 nm, which corresponds to the interplanar distance of (1 1 0) planes SnO_2_ [16,30,31]. These data infer that the calculated average particle sizes from XRD, SEM and TEM measurements have a consistent value. The particle size obtained in this work is at the average and within the range found using other plants extracts, as presented in Table 1. Many researches have found the spherical form of nanoparticles to be within the range of 2–50 nm, except concerning *Ficus carica* with a size of 128 nm. The form and particle size are strongly influenced by several factors such as composition of the extract, reaction procedure and temperature of sintering [32]. 

The different compositions of secondary metabolites govern the bioreduction of Sn^2+^ and furthermore affect the particle’s aggregation after the sintering process, which has also been reported in the green syntheses of ZnO, Fe_2_O_3_ and SnO_2_ nanoparticles [28,32,38,39]. 

### 3.2. Optical Properties of SnO_2_ NPs 

**Figure 8:**
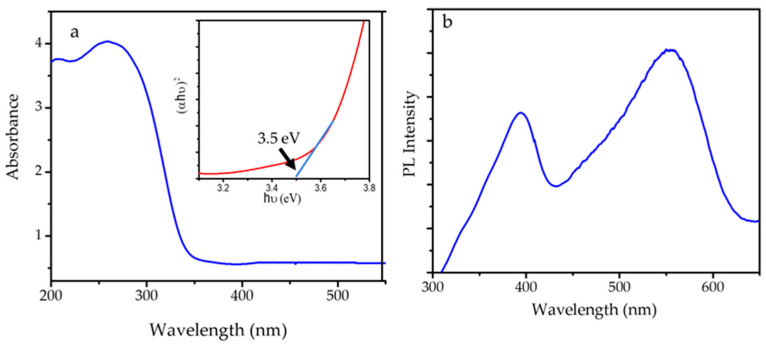
(**a**) UV-DRS spectrum, and (**b**) PL spectrum of SnO_2_ NPs.

The optical properties of SnO_2_ NPs were studied by UV-DRS and photoluminescence spectroscopy analyses, and the spectra are presented in Figure 8. The band gap of SnO_2_ NPs was evaluated according to the UV-DRS spectrum with the following Equation (2):

(αhν)^2^=A(hν − Eg) (3)

where α, h, ν, E_g_ and A are the absorption coefficient, Planck constant, light frequency, bandgap energy and Tauc constant, respectively. The extrapolation of (α h ν)^2^ versus hν to zero gives the band gap energy of 3.5 eV. This value lies within the range of 3.1–3.9 eV, as mentioned in the literatures, and is comparable to the band gap energy of SnO_2_ NPs synthesized using the hydrothermal method [17,40,41]. The photoluminescence (PL) spectrum was recorded with varying excitation wavelengths (l = 360~380 nm), and the spectrum of SnO_2_ NPs showed an excitation wavelength of 396 and 550 nm, of which the peak at 550 nm is attributed to 6A1→4T1(4G) ligand field transition of Sn^4+^. The data represents that SnO_2_ NPs have the capability to interact with photons in either the UV or the visible region. 

### 3.3. Photocatalytic Activity 

**Figure 9:**
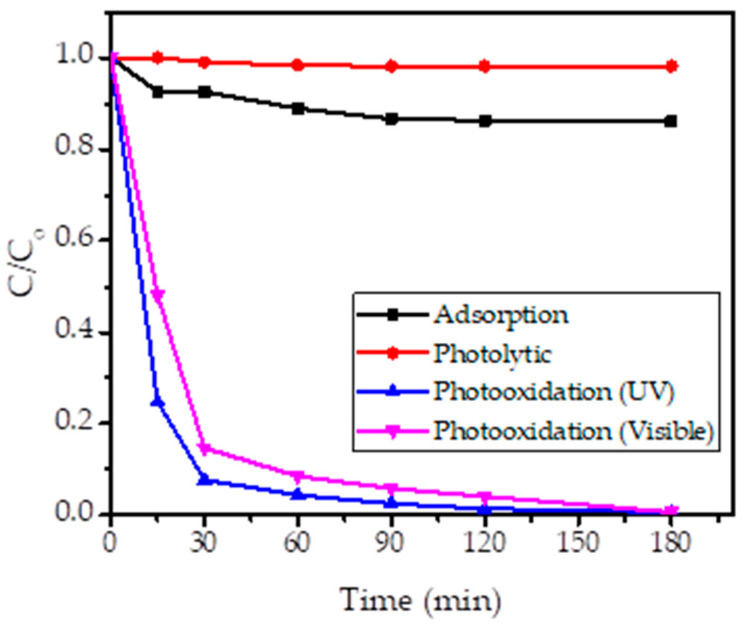
Kinetics of BPB decolorization by varied treatments.

Figure 9 shows the kinetics plot of BPB photooxidation under UV and visible light irradiation in comparison with blank experiments consisting of adsorption and photolytic treatments. The adsorption treatment was the treatment with the addition of SnO_2_ NPs in the solution without any light exposure, while the photolytic experiment was the UV light exposure to the solution with the addition of H_2_O_2_ in the absence of photocatalysts. It can be seen that the photocatalytic and photooxidation experiments significantly reduced BPB concentration attributed to the decolorization from the initial step of the reaction, while the blank experiments reveal insignificant decolorization. The decolorization of BPB reaches less than 5% with respect to the initial concentration by photolytic treatments; meanwhile, there is about 15% reduction concerning the adsorption experiment for 120 min. The decolorization for the adsorption experiment is attributed to the role of SnO_2_ NPs in providing surface interactions with the BPB molecules, as has been reported in previous experiments of dye adsorption using SnO_2_ and SnO_2_ NPs [42,43]. The photolytic mechanism depends on the capability of light to produce radicals from the homolytic cleavage of H_2_O_2_ molecules and, from the experiment, it was conclusively obtained that the reaction went slower compared to the formation of radicals over the catalytic mechanism. 

It was also seen that the use of UV light illumination demonstrated a faster removal. While the PL intensity of SnO_2_ NPs supports the photoactivity within the visible light region, according to the band gap energy value which lays within the UV light range, the photon interaction with UV light produced the radicals for further propagation reactions. 

### 3.4. Identification on Oxidation Mechanism 

**Figure 10:**
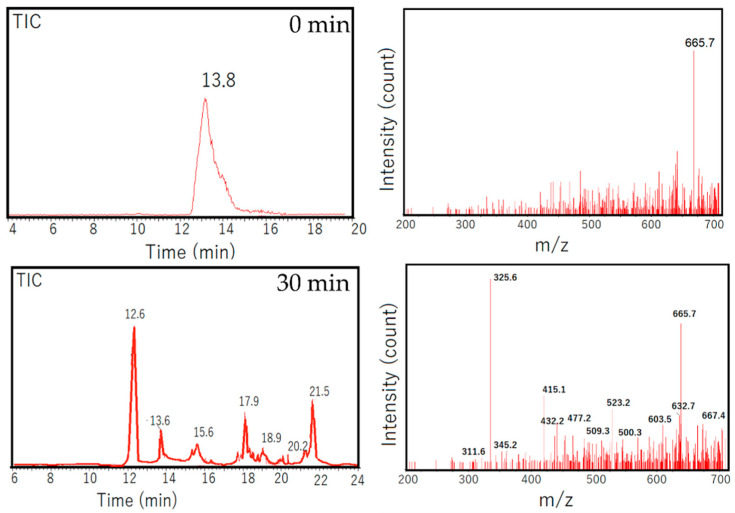
LCMS of initial and treated solutions.

For the verification of the occurrence of the oxidation mechanism, LCMS analyses were performed. Results from LCMS analysis presented in Figure 10 validated the degradation mechanism, concerning which the chromatogram of the initial solution demonstrated a single peak at 12.8 min and later changed, displaying other peaks in the treated solution, indicating that the products degraded [44]. In addition, the MS spectra of the initial and treated solutions suggested the predicted reaction intermediates from the presence of *m*/*z* = 667.4, 665.7, 632.7, 603.5, 500.3, 523.2, 415.1 and 325.6. The degradation mechanism and products of azo dyes have been extensively discussed, and refer to the existence of the peak *m*/*z* = 667.4, which is higher compared to the *m*/*z* reflecting the presence of BPB (665.7), indicating the addition of the radical as the first step of the propagation [45,46].

**Figure 11:**
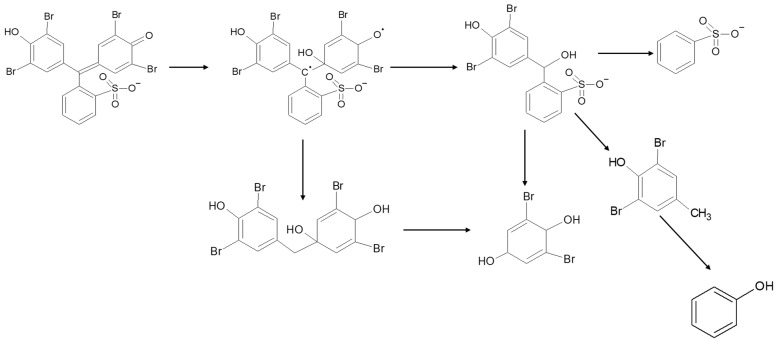
Proposed degradation mechanism of BPB by photooxidation.

Referring to these MS peaks, the possible degradation mechanism can be seen in Figure 11. A similar mechanism was reported for BPB removal over CuO-nano-clinoptilolite and BPB degradation by sono-catalytic treatment using SnO_2_/montmorillonite [47,48]. 

These data revealed the role of SnO_2_ NPs to accelerate the radical formation and propagation steps by the following mechanism:

SnO_2_ NPs + hν→SnO_2_ NPs* + h_vb_^+^ + e_cb_^−^

H_2_O + h^+^→H^+^ + OH•

OH^−^ + h^+^→OH•

O_2_ + e^−^→O_2_^−^

O_2_ + 2H^+^ + 2e^−^→H_2_O_2_

H_2_O_2_ + e^−^→OH• + OH^−^

BPB + •OH + O_2_→degradation products→→→CO_2_ + H_2_O

**Figure 12:**
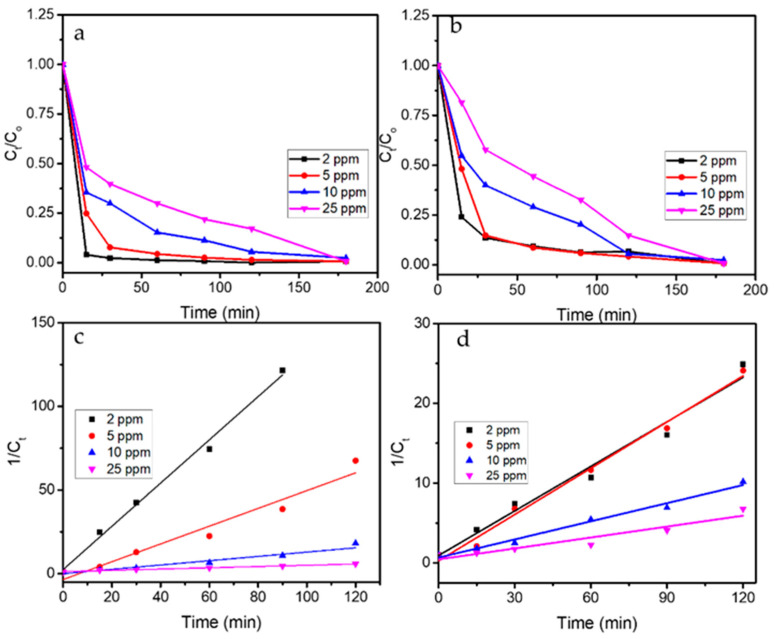
(**a**) Kinetics plots of BPB photooxidation under UV light. (**b**) Kinetics plots of BPB photooxidation under visible light. (**c**) Pseudo-second-order plot of BPB photooxidation under UV light. (**d**) Pseudo-second-order plot of BPB photooxidation under visible light.

Furthermore, the kinetics plots of photooxidation reactions from varied concentrations are depicted in Figure 12. Generally speaking, the compared data are consistent with the trends of the higher degradation rate which were achieved under UV exposure rather than the use of visible light. The kinetics evaluation on these data suggests that the reactions obey the pseudo-second order kinetics with reference to following equation (Equation (4)):

(4) $$\frac{1}{C_{t}}=kt+\frac{1}{C_{0}}$$

where *C*_0_ and *C_t_* are initial concentration and concentration of BPB at time *t*, and *k* is kinetics constant. 

The fitness of the data concerning pseudo-second-order kinetics is similar to the kinetics of BPB photocatalytic degradation over SnO_2_ synthesized using red spinach [37], which also represented the effect of the initial concentration of dye concerning the reaction rate. The kinetics parameters and DE values are presented in Table 2. 

### 3.5. Effect of Scavenger 

**Figure 13:**
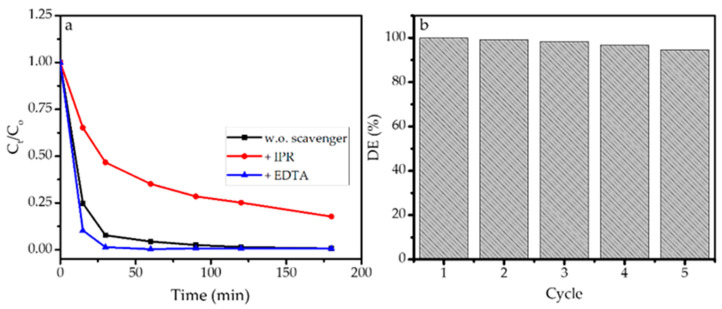
(**a**) Kinetics plot of BPB photooxidation in the absence and presence of scavenger. (**b**) DE (%) of photooxidation process at 1st until 5th cycles.

To study the role of ∙OH in photooxidation, the effect of scavenging agents consisting of EDTA as a hole scavenger and isopropanol as the radical’s scavenger on kinetics of BPB removal was examined. Figure 13a shows that isopropanol significantly suppressed the degradation of BPB as slower C/C_0_ reduction occurred. Isopropanol traps the radicals formed by the excitation produced by the interaction between SnO_2_ NPs and light so the propagation steps for producing further oxidation reaction are diminished. In contrast, the addition of EDTA enhanced the photooxidation with about 10% BPB reduction being observed at the initial part of the reaction. The kinetics reflected that EDTA inhibits the recombination of electrons and holes so more electrons can migrate to the surface of the SnO_2_ NPs and further react with O_2_ to form HOO• [48]. 

### 3.6. Reusability of Photocatalysts 

The reusability of photocatalysts is an important character which governs the applicability for further scale. In order to check the reusability of photocatalysts, the spent photocatalyst was filtered and recycled by washing with ethanol, followed by drying at 200 °C for 2 h. A mass loss of about 5–10% wt. occurred due to technical steps such as incompleteness in recovery by filtration. The degradation efficiency of recycled photocatalysts compared to fresh ones is presented in a chart in Figure 13b. It is seen that DE remained stable in the range of 98.52–94.60% until the fifth cycle, which means that the reduced activity was no more than 10%. This performance is attributed to the stability of the nanoparticles which related to the maintained physicochemical characteristics concerning its role in the photooxidation mechanism. The reusability of the SnO_2_ is comparable with magnetite nanoparticles and SnO_2_ dispersed onto montmorillonite [47], but the crucial factor of recovery after the use of photocatalyst needs to be addressed for improvement in industrial or scaled-up levels.

### 3.7. Antibacterial Activity 

**Figure 14:**
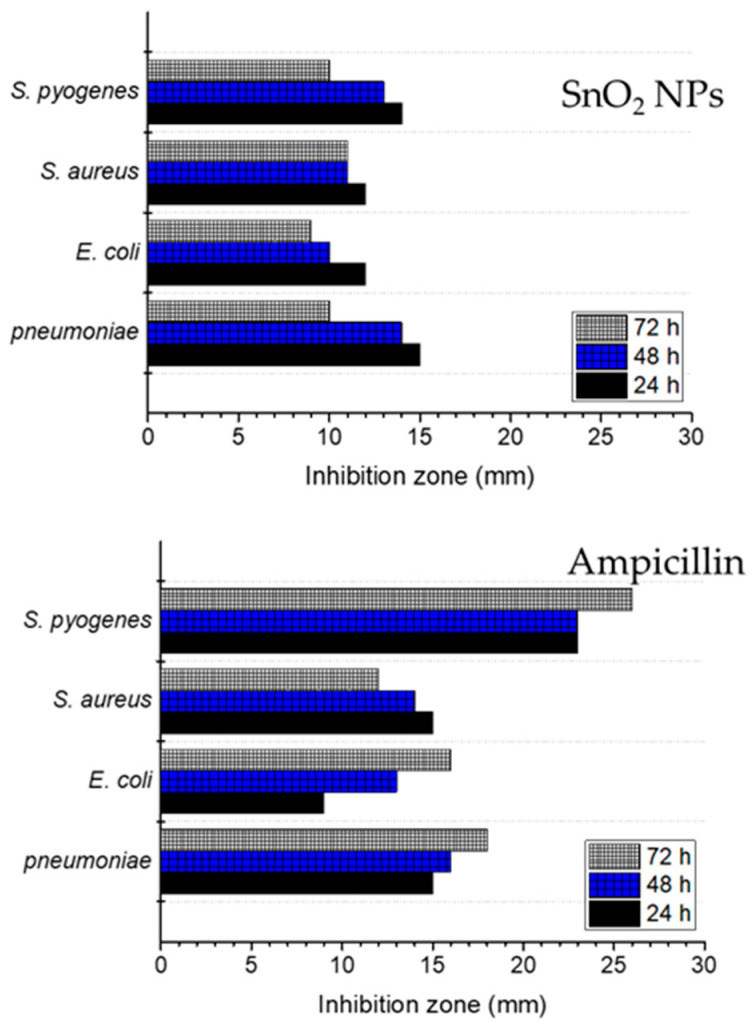
Inhibition zone of SnO_2_ NPs against tested bacteria, in comparison with ampicillin.

Disk diffusion assay was performed to analyze the inhibition zone of SnO_2_ NPs against *K. pneumoniae*, *E. coli*, *S. aureus* and *S. pyogenes*. Figure 14 shows the inhibition zone diameter for each test in comparison with ampicillin as the positive control. It is also found that the inhibition zone reduced at prolonged incubation times for 48 and 72 h, suggesting that the nanoparticles’ capability is more for inhibiting rather than killing the tested bacteria. 

The data show that the inhibition zone diameters for all tested bacteria are smaller compared to those of ampicillin as positive control. The different mechanism is a known factor in this, and ampicillin is a well-known pharmaceutical, being an antimicrobial agent. In more detail concerning SnO_2_ NPs, higher antibacterial effects against *K. pneumoniae* and *S. pyogenes*, which are gram-positive bacteria, compared to those of *E. coli* and *S. aureus*, are demonstrated. With regard to a previous study on the surface interaction among nanoparticles and the bacteria, the stronger surface interaction of nanoparticles with gram-positive bacteria is due to a partially less negative surface potential of the bacteria. 

**Figure 15:**
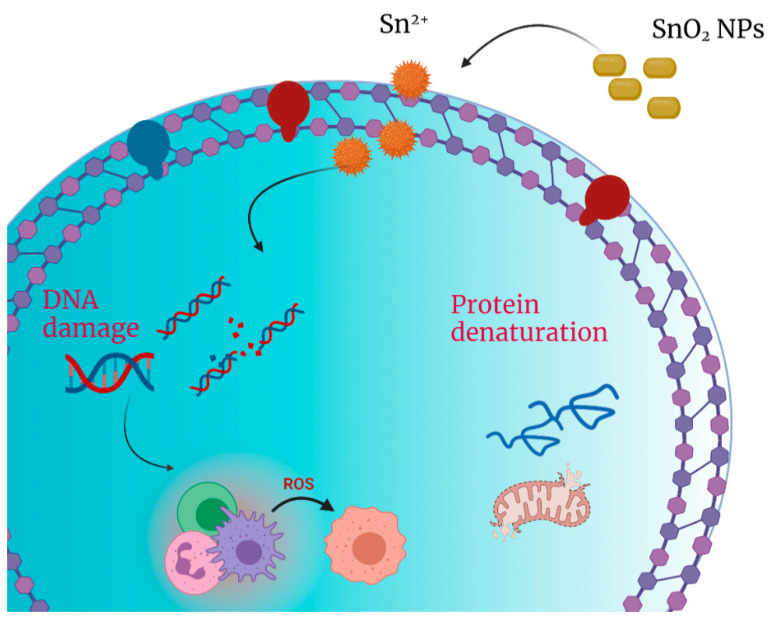
Schematic representation of bacteria inactivation mechanism.

Metal oxide nanoparticles tend to have a negative zeta potential, which easily interacts with this surface potential according to the electrostatic force equilibrium. In contrast, the more negative the surface potential of the gram-negative bacteria, the less the surface interaction [49,50,51,52]. Ions can be released from the interaction between SnO_2_ NPs with the cell wall leading to the generation of reactive oxygen species (ROS) on the surface of the nanoparticles. The DNA damage causing cell destruction is a further step for bacteria inactivation. Figure 15 depicts the schematic representation of the mechanism [53].

The inhibition zone which identified the antibacterial activity of SnO_2_ NPs in this study was comparable to, and even greater than, the SnO_2_ NPs observed in other studies. For example, the activity against *E coli* was higher compared to that demonstrated by the SnO_2_ NP synthesized co-precipitation method using DNA as the capping agent [54], and was also higher compared to SnO_2_ NPs synthesized using *Vitex altissima* [52]. The inhibition zone of 12 mm in this research was achieved with a concentration of 200 μg/mL, higher than the inhibition zone from the DNA-synthesized SnO_2_ NPs (10 mm) and *Vitex altissima*-mediated SnO_2_ NPs (1.1 mm). Higher activity was also demonstrated against *S. aureus* and *K. pneumoniae* compared to *Stevia rebaudiana*-mediated SnO_2_ NPs [55]. The particle size of *Stevia rebaudiana*-mediated SnO_2_ NPs ranged from 20 to 30 nm, suggesting that the higher antibacterial activity in this work is strongly correlated with smaller particle size. Smaller particle size enables the intrusion of nanoparticles into the cell wall to further destroy the structure of the bacteria [32,56,57,58]. Generally speaking, the synthesized SnO_2_ NPs have pronounced antibacterial activity concerning tested gram-negative and gram-positive bacteria, so it has potentially further developed for environmental and biomedical applications.

## 4. Conclusions 

This research, the first ever study on the synthesis of SnO_2_ nanoparticles using *Pometia pinnata* leaf extract, demonstrated the flower-like structure of nanoparticles as a single SnO_2_ tetragonal phase. TEM analysis proved the nanoparticles had uniform spherical shapes with size ranging from 8 to 20 nm, which was confirmed with XRD analysis, finding a crystallite size of 18.2 nm. Optical study of the nanoparticles showed that the band gap energy of SnO_2_ nanoparticles is 3.5 eV, contributing to the photocatalytic activity in bromophenol blue photooxidation. Kinetics study revealed the fitness of bromophenol blue photooxidation with second-order kinetics by either UV or visible light exposure. Degradation efficiencies ranging from 85 to 99% were found with dependency toward an initial concentration of BPB and the use of a light source. The antibacterial activity evaluation of SnO_2_ nanoparticles indicated an inhibition capability against *Klebsiella pneumoniae*, *Escherichia coli Staphylococcus aureus* and *Streptococcus pyogenes* which is comparable and even higher in comparison with SnO_2_ nanoparticles reported in previous studies. 

## Figures and Tables

**Table 1:** Comparison of morphologies and particle size of SnO_2_ NPs synthesized using various plant extracts.

Bioreductor.	Morphology	Particle Size (nm)	References
*Camellia sinensis* flower extract	spherical	5–30	[32]
*Vernonia amygdalina* leaf extract	nanorod	6.45	[33]
*Menta spicata* leaf extract	nanorod	7.35	[33]
*Actinidia deliciosa* (Kiwi) peel extract.	spherical	20	[34]
*Galaxaura elongata*	spherical	35	[35]
*Ficus Carica leaf*	spherical	128	[36]
*Calotropis gigantea*	irregular	35	[27]
*Vitex altissima (L.)* Leaf Extract	spherical	20	[27]
Red spinach leaf extract	Spherical	20–40	[37]

**Table 2:** Kinetics constant and DE of BPB photooxidation at varied initial concentrations.

Initial Concentration	Light	R^2^ of the Second Order Kinetics	Kinetics Constant k (L/mg.min)	DE at 120 min (%)
2	UV	0.997	3.41	99.93
5	UV	0.993	0.72	98.52
10	UV	0.995	0.60	94.50
25	UV	0.994	0.21	92.85
2	Visible	0.994	0.67	93.29
5	Visible	0.996	0.63	95.85
10	Visible	0.996	0.60	94.50
25	Visible	0.996	0.21	85.16

## Data Availability

This study did not report any data.
